# Association between healthy lifestyle and the occurrence of cardiometabolic multimorbidity in hypertensive patients: a prospective cohort study of UK Biobank

**DOI:** 10.1186/s12933-022-01632-3

**Published:** 2022-10-01

**Authors:** Hejian Xie, Jinchen Li, Xuanmeng Zhu, Jing Li, Jinghua Yin, Tianqi Ma, Yi Luo, Lingfang He, Yongping Bai, Guogang Zhang, Xunjie Cheng, Chuanchang Li

**Affiliations:** 1 Department of Geriatric Medicine, Center of Coronary Circulation, Xiangya Hospital, Central South University, Xiangya Road 87#, Changsha, 410008 China; 2 National Clinical Research Center for Geriatric Disorders, Xiangya Hospital, Central South University, Changsha, China; 3 Department of Cardiovascular Medicine, The Third Xiangya Hospital, Central South University, Changsha, China; 4 Department of Cardiovascular Medicine, Xiangya Hospital, Central South University, Changsha, China

**Keywords:** Cardiometabolic multimorbidity, Healthy lifestyle, Hypertension, Coronary heart disease, Stroke, Diabetes mellitus

## Abstract

**Background:**

Cardiometabolic multimorbidity (CMM) is becoming increasingly common in patients with hypertension, and it is well established that healthy lifestyle plays a key role in the prevention of hypertension. However, the association between combined lifestyle factors and CMM in patients with hypertension is uncertain.

**Methods:**

This prospective analysis included the data (obtained from the UK biobank) of participants with hypertension who did not have coronary heart disease (CHD), stroke, or diabetes. The outcome was the occurrence of CMM, defined as ≥ 1 disease of CHD, stroke, and diabetes that occurred in participants with hypertension. Four lifestyle factors (smoking, alcohol consumption, diet, and physical activity) were assessed using a weighted healthy lifestyle score, and participants were divided into four groups: the very unhealthy, unhealthy, healthy, and very healthy groups. The flexible parameter Royston-Parmar proportional hazard model was used to estimate hazard ratios (HRs) between lifestyles and CMM, as well as the difference in CMM-free life expectancy.

**Results:**

During a median follow-up of 12.2 years, 9812 (18.4%) of the 53,397 hypertensive patients occurred CMM. Compared with the very unhealthy group, the very healthy group had a 41% reduction in the risk for CMM in hypertensive patients and a 32–50% reduction in the risk for specific cardiometabolic diseases such as CHD, stroke, and diabetes. For each lifestyle factor, non-smoking had the greatest protective effect against CMM (HR: 0.64, 95% confidence interval (CI) 0.60–0.68). A lifestyle combining multiple healthy factors extended CMM-free life expectancy (e.g., six years longer at age 45 years for participants in the very healthy group).

**Conclusions:**

Combined healthy lifestyle factors were associated with a lower risk for CMM in hypertensive patients. This suggests that combined healthy lifestyle should be supported to decrease disease burden.

**Supplementary Information:**

The online version contains supplementary material available at 10.1186/s12933-022-01632-3.

## Introduction

With the increasing proportion of aging people in the global population, multimorbidity is becoming a global public health concern owing to increased disability and mortality, reduced quality of life, and increased disease burden [1–4]. Cardiometabolic multimorbidity (CMM), defined as the coexistence of two or more cardiometabolic diseases including hypertension, coronary heart diseases (CHD), stroke, and diabetes, is one of the most common and severe multimorbidity [1, 5]. Compared with patients without cardiometabolic diseases, patients with CMM have a 3.7–6.9 times higher risk of all-cause mortality and a 12–15 year reduction in life expectancy at age 60 [1].

Hypertension is the most common component of CMM [6]. In the UK Biobank database, 70%, 64%, 59%, and 57% of patients diagnosed with chronic kidney disease, diabetes, angina, and stroke, respectively, were also diagnosed with hypertension [6]. In addition, one in four patients with hypertension also develop CMM [2]. After comorbidity with diabetes and cardiovascular disease, the risk of all-cause mortality in hypertensive patients increases significantly from 7 to 30% and 136%, respectively [5]. Therefore, identifying effective strategies for CMM prevention in hypertensive patients is essential.

Lifestyle interventions such as non-smoking, non-excessive alcohol consumption, a healthy diet, and regular physical activity have been consistently recommended by many guidelines and studies for the prevention and management of hypertension [7–9]. However, existing data showed that healthy lifestyles have not been well adopted by patients with hypertension. A study showed that only 1.7% of hypertensive patients reported adherence to five healthy lifestyle factors—non-smoking, normal weight, limited alcohol consumption, healthy diet, and regular physical activity [10]. While previous studies showed that combined healthy lifestyle factors reduced the incidence of cardiovascular disease, CMM, and mortality in the general population [11, 12]. It is inappropriate to directly assume the same implications for the occurrence of CMM in patients already diagnosed with hypertension, as single risk factors play distinct roles at varying stages of the disease [13]. Recently, a study showed that compared with 0 low-risk lifestyle factors, adhering to 5–6 low-risk lifestyle factors reduced the risk of type 2 diabetes by 86% in patients with hypertension [14]. These factors included normal body mass index (BMI), non-smoking, moderate alcohol consumption, regular physical activity, healthy diet, and healthy sleep patterns. However, as different lifestyle factors contribute unequally to disease occurrence, using a summation method to assess lifestyles may not accurately determine their real-world impact on human health [15, 16]. At present, the effect of combined lifestyle factors on the occurrence of CMM in hypertensive patients has not been investigated. Revealing the relationship between combined lifestyle factors and CMM is crucial for CMM prevention in hypertensive patients.

Therefore, this study aimed to explore the association between combined healthy lifestyle factors and the occurrence of CMM in patients with hypertension. We also calculated the differences in CMM-free life expectancy in the various lifestyle groups.

## Methods

### Study population

**Fig. 1:**
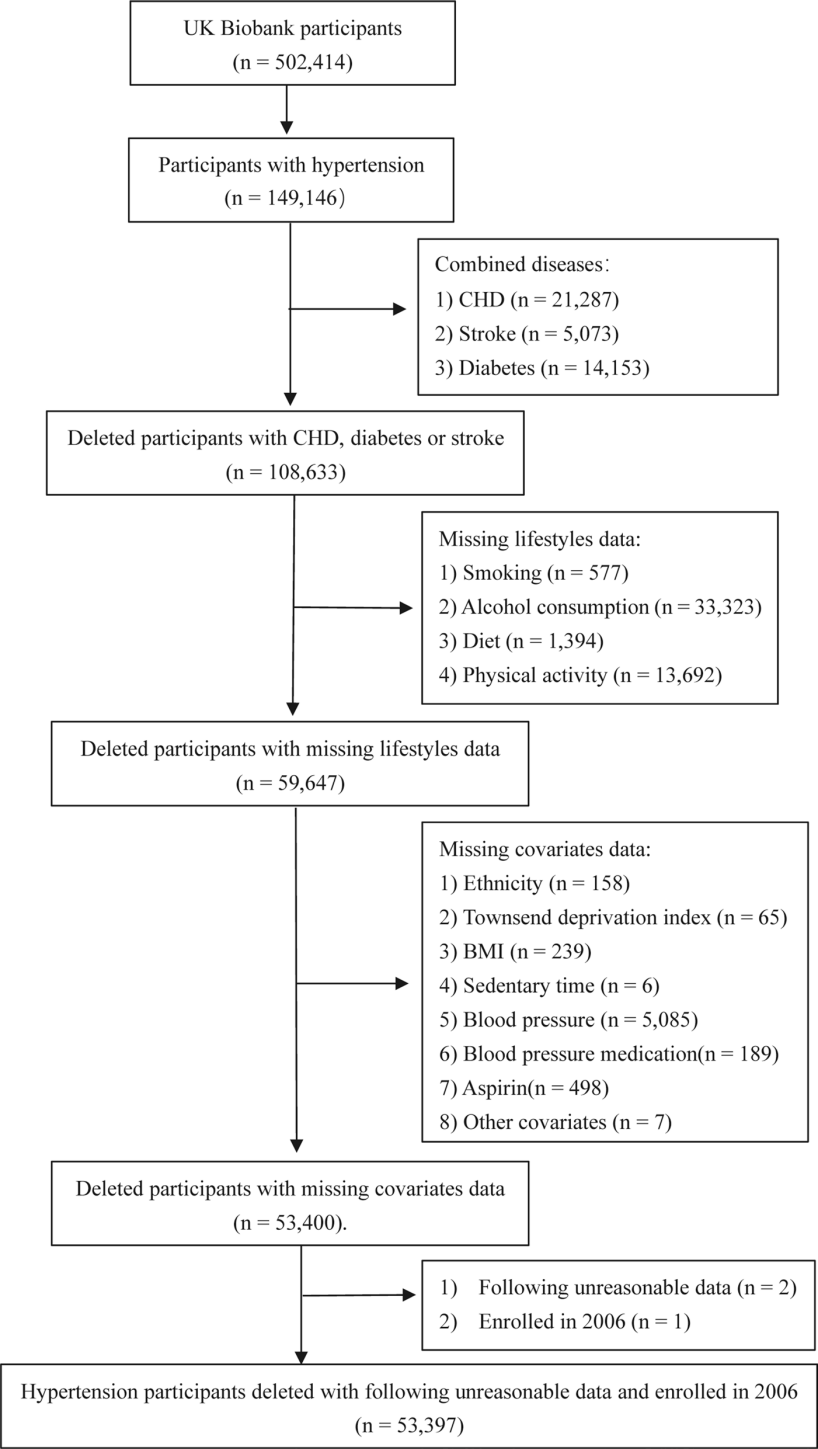
Screening flowchart for baseline hypertensive population. Other covariates included missing the occurrence age of cardiometabolic multimorbidity. The only participant enrolled for the year (this occurred in 2006, resulting in a large difference between the groups in the year of enrolment, so it was removed). *CHD* coronary heart disease, *BMI* body mass index

This study used data from the UK Biobank (Application Number 76118), which recruited over 500,000 middle-aged (aged 37–74 years) participants from 22 assessment centers in England, Scotland, and Wales between 2006 and 2010. After obtaining written informed consent from the participants, researchers collected health information through interviews, questionnaires, health records, anthropometry, biological samples (blood, urine, and saliva), and images [16]. The National Research Ethics Committee of the National Health Service approved this data collection (UK). This study specifically included UK Biobank data of patients with hypertension (n = 149,146). Hypertension at baseline was ascertained according to the information in self-reported history, physician diagnosis, medication history, and inpatient records of diagnoses (Additional file 1: Table S1). We also established the following exclusion criteria for participants: (1) CHD, diabetes or stroke at baseline (n = 40,513); (2) missing information on target lifestyle factors (n = 48,986); (3) missing covariates data (n = 6247); (4) the following unreasonable data (n = 2); and (5) being the only participant enrolled for the year (this occurred in 2006, resulting in a large difference between the groups in the year of enrolment, so it was removed). Ultimately, 53,397 participants remained eligible (Fig. 1, Additional file 1: Table S1).

### Healthy lifestyle

We used the following lifestyle factors, selected according to international guidelines [16–20]: smoking, alcohol consumption, diet, and physical activity.

Lifestyle information was obtained from the UK Biobank touchscreen questionnaires. Smoking was divided into two categories at baseline: current and non-current smoking [16]. For alcohol consumption, we measured weekly consumption of red or white wine, beer, cider, spirits, liqueurs, and fortified wine, and converted them into equivalent standard units according to UK National Health Service guidelines [17] and Office for National Statistics survey data [18] (Additional file 1: Text S1). Participants were grouped into those with excessive drinking (≥ 14 units per week) and those with non-excessive drinking (< 14 units per week) [16]. A healthy diet was determined based on a healthy diet score adopted from the American Heart Association guidelines [19], which assigned one point for more than 2 of the following three items, otherwise 0 points: (1) Total fruit and vegetable intake: ≥ 4.5 pieces or servings per day (1 serving being 3 tablespoons); (2) Total fish intake ≥ 2 times per week; (3) Red meat intake ≤ 5 times per week and processed meat intake ≤ 2 times per week. For physical activity, researchers used touchscreen questionnaires to ask participants about the type, frequency, and duration of exercise they engaged in. We then calculated the total duration of exercise to assess whether participants met 150 minutes of walking or moderate activity per week or 75 minutes of vigorous activity requirement stipulated in the 2019 UK Physical Activity Guidelines [20]. Those who met the exercise standards were classified as a regular physical activity group.

We then calculated the weighted healthy lifestyle score by combining the four risk factors. Additional details of this calculation are provided in the statistical analysis section.

### Outcome assessment

The outcome measured in this study was the occurrence of CMM in hypertensive patients. The UK Biobank collected participants’ data from self-reported diseases diagnosed by doctors and hospital inpatient records. Diagnoses were coded according to the International Classification of Disease versions 9 and 10 and the Office of Population Censuses and Surveys Classification of Interventions and Procedures version 4. CMM is commonly defined as the coexistence of two or more cardiometabolic diseases; however for this study, CMM was defined as the occurrence of ≥ 1 of the following: CHD (myocardial infarction or angina), stroke (ischaemic stroke, cerebral haemorrhage or subarachnoid haemorrhage), and diabetes (Additional file 1: Table S1). The date of CMM was the earliest date of occurrence for any of the three cardiometabolic diseases.

### Other covariates

Other relevant covariates were also extracted including sex, ethnicity (white or non-white), Townsend deprivation index (mainly included index of multiple deprivations, education score, crime score, employment score, health score, income score, housing score, living environment score), BMI (weight kilograms divided by height meters squared), sedentary time (time spent watching TV, using the computer, and driving), antihypertensive and lipid-lowering drugs use, aspirin use, total energy intake, and the year the patients attended the assessment centre.

### Statistical analysis

Baseline characteristics stratified by the lifestyle score categories are presented as mean and standard deviation (continuous variables) or numbers and percentages (categorical variables).

There were potential differences in the association between each healthy lifestyle and the risk for CMM. Therefore, we calculated weighted healthy lifestyle scores using the flexible parameter Royston–Parmar proportional hazard model [21] to estimate the β coefficient of each healthy lifestyle factor. The model included all four lifestyle factors and the occurrence of CMM as the outcome. The four lifestyle factors were classified into two categories; one point was assigned for a regular healthy lifestyle as described earlier, otherwise 0 points were assigned. Next, we calculated the standardised weighted β coefficient thus: standardised weighted β coefficient = the β coefficient of each healthy lifestyle factor/sum of the β coefficients (Additional file 1: Text S2). Then, we multiplied the binary lifestyle variables of each participant using the standardised weighted β coefficient and then summed and grouped participants into four ordered categories [16]: very unhealthy (0 ≤ scores < 0.25, reference group), unhealthy (0.25 ≤ scores < 0.50), healthy (0.50 ≤ scores < 0.75), and very healthy (0.75 ≤ scores ≤ 1). We calculated the hazard ratios (HRs) and corresponding 95% confidence intervals (CIs) between the aggregated healthy lifestyle and CMM based on the flexible parameter Royston-Parmar proportional hazard model with age as the time scale, followed by assessment of the role of each individual lifestyle. Similarly, we adopted the same method to calculate the HRs and corresponding 95% CIs between combined healthy lifestyles and a specific CMM (CHD, stroke, and diabetes). The confounding factors were adjusted for in the three models. Model 1 was adjusted for sex and ethnicity; Model 2 was additionally adjusted for Townsend deprivation index, BMI, and sedentary time; and Model 3 was additionally adjusted for antihypertensives drugs use, lipid-lowering drugs use, aspirin use, and the year of attending the assessment centre.

We used the flexible parameter Royston–Parmar proportional hazard model to calculate the differences in CMM-free life expectancy in three steps. First, we calculated the mean CMM-free life expectancy for participants who survived to ages 45–100 years (intervals of each year). We then computed the curves of the CMM-free life expectancy for the four lifestyle score categories and obtained the restricted mean survival time (RMST) [22], which indicates the area under a survival curve between ages 45 to 100 years of age. Finally, we compared the very unhealthy group (reference group) with the unhealthy, healthy, and very healthy groups, and calculated the difference in CMM-free life expectancy as the difference between every two RMSTs. The difference in CMM-free life expectancy was adjusted according to Model 3, and the number of knots used in the Royston-Parmar proportional hazard model was four.

### Subgroup analysis

We performed subgroup analysis stratified by sex (male, female), ethnicity (white, non-white), BMI (< 30, ≥ 30), Townsend deprivation index (≤ 0, > 0), and sedentary time (< 5 h,  ≥ 5 h) and use of antihypertensive drugs, lipid-lowering drugs, and aspirin.

### Sensitivity analysis

To verify the stability of our results, seven sensitivity analyses were conducted. First, we excluded 357 participants who had either died or occurred CMM within the first two years of follow-up to examine whether the results were affected by reverse causation bias. Second, we excluded 5859 participants with new-onset hypertension within 1 year, as these patients may have changed any unhealthy lifestyle factors after receiving the diagnosis of hypertension. Third, the researchers asked the participants how many days in each week they walked or engaged in moderate or vigorous physical activity for more than 10 minutes, but some participants did not provide a duration. We recorded 10 minutes of physical activity each time for those who answered “Do not know” or “Prefer not to answer.” Fourth, we used a random one-third of the dataset to derive the β coefficient and reperformed our analyses based on the remaining two-thirds of participants [16] (Additional file 1: Text S2). Fifth, we summed up the four lifestyle scores, ranging from 0 to 4. Participants who scored 0 and 1 were categorised into the very unhealthy group, while those who scored 2, 3, and 4 were categorised into the unhealthy, healthy, and very healthy groups, respectively. We calculated the HRs and corresponding 95% CIs between the combined healthy lifestyle and CMM with the flexible parameter Royston–Parmar proportional hazard model using age as the time scale in Model 3. Then, using the same method with the lifestyle score as a continuous variable, HRs and corresponding 95% CIs were calculated. Sixth, we included total energy intake as a covariate in Model 3 for adjustment. A total of 45,033 individuals were excluded because of missing data on total energy intake. The remaining 8364 patients with hypertension were analysed using the same method. Seventh, we validated our findings by shortening the follow-up period to 9.0 years to account for potential changes in lifestyle factors and variables that may result from long-term follow-up.

R version 4.0.5 and Stata version 17.0 were used to conduct data analyses, and a two-side *P* value < 0.05 was considered statistically significant.

## Results

### Baseline characteristics

Baseline characteristics of hypertensive participants based on healthy lifestyle scores are shown in Table 1. The median (range) age of 53,397 hypertensive patients was 61.0 (40.0–71.1) years, and most participants were white (97.3%). All participants were divided into four groups based on healthy lifestyle scores, with 2505 grouped as very unhealthy, 4242 as unhealthy, 13,955 as healthy, and 32,695 as very healthy. Compared to the very unhealthy group, participants with higher lifestyle scores were more likely to be older, have a wealthy economy, have less sedentary time, and use antihypertensive drugs. Sex, ethnicity, BMI, blood pressure, and use of lipid-lowering drugs and aspirin were similar across healthy lifestyle score groups.

**Table 1:** Baseline characteristics of hypertension participants by healthy lifestyle score

	Healthy lifestyle score			
	Very unhealthy	Unhealthy	Healthy	Very healthy
No. (%) of participants	2505 (4.7)	4242 (7.9)	13,955 (21.6)	32,695 (61.2)
Age at survey, mean (SD), years	56.46 (7.69)	57.87 (7.34)	58.84 (7.16)	60.19 (6.85)
Sex				
Women, No. (%)	626 (25.0)	1292 (30.5)	4048 (29.0)	16,887 (51.7)
Men, No. (%)	1879 (75.0)	2950 (69.5)	9907 (71.0)	15,808 (48.3)
Ethnicity				
Non-white, No. (%)	94 (3.8)	132 (3.1)	255 (1.8)	930 (2.8)
White, No. (%)	2411 (96.2)	4110 (96.9)	13,700 (98.2)	31,766 (97.2)
Townsend deprivation index, mean (SD)^a^	− 0.16 (3.43)	− 1.14 (3.17)	− 1.57 (2.91)	− 1.82 (2.77)
BMI, mean (SD), kg/m^2^	28.13 (4.67)	29.20 (4.85)	29.04 (4.48)	28.00 (4.40)
Sedentary time, mean (SD)^b^, h	5.65 (2.87)	5.28 (2.52)	4.91 (2.33)	4.66 (2.11)
Systolic blood pressure, mean (SD), mmHg	147.25 (18.58)	147.49 (17.46)	148.94 (17.45)	148.85 (17.89)
Diastolic blood pressure, mean (SD), mmHg	88.58 (10.67)	88.70 (10.11)	88.65 (9.79)	87.27 (9.71)
Use of lipid-lowering drugs, No. (%)	666 ( 26.6)	1200 (28.3)	3950 (28.3)	8591 (26.3)
Use of antihypertensive, No. (%)	1389 (55.4)	2613 (61.6)	8765 (62.8)	20,993 ( 64.2)
Use of aspirin, No. (%)	446 (17.8)	833 (19.6)	2451 (17.6)	5564 (17.0)
Healthy lifestyle factors^c^				
Regular physical activity, No. (%)	1558 (62.2)	1609 (37.9)	9842 (70.5)	30,404 ( 93.0)
Not currently smoking, No. (%)	0 (0.0)	2510 (59.2)	13,452 (96.4)	32,696 (100.0)
Healthy diet, No. (%)	264 (10.5)	1234 (29.1)	2562 (18.4)	26,107 (79.9)
Non-excessive drinking, No. (%)	200 (8.0)	621 (14.6)	2557 (18.3)	21,074 (64.5)

Continuous variables are shown by mean and standard deviation, categorical variables are shown by numbers and percentages

*BMI* body mass index

^a^Townsend deprivation index: Townsend deprivation index was used as a measure of socioeconomic status, which mainly included index of multiple deprivations, education score, crime score, employment score, health score, income score, housing score, living environment score

^b^Sedentary time: The total self-reported hours spent watching television, using the computer, or driving

^c^Regular physical activity: 150 minutes of walking or moderate activity per week or 75 minutes of vigorous activity; Non-excessive drinking:we collected weekly consumption of red wine, white wine or champagne, beer or cider, spirits or liqueurs, and fortified wine and converted them into equivalent standard units according to National Health Service guidelines and the Office for National Statistics survey data (Additional file 1: Text S1). The participants were grouped as excessive drinking ( ≥ 14 units per week) or non-excessive drinking (< 14 units per week). Healthy diet: (1) Total fruit and vegetable intake: ≥ 4.5 pieces or servings per day (1 serving being 3 tablespoons); (2) Total fish take ≥ 2 times per week; (3) Red meat intake ≤ 5 times per week and processed meat intake ≤ 2 times per week. The healthy diet score was 1 point for more than 2 of the following three items, otherwise 0 points

### Healthy lifestyle and CMM

During median follow-up 12.2 years, 9812 (18.4%) of the 53,397 hypertensive patients occurred CMM, 5593 (10.5%) of whom had CHD, 2572 (4.8%) had stroke, and 3083 (5.8%) had diabetes.

**Fig. 2:**
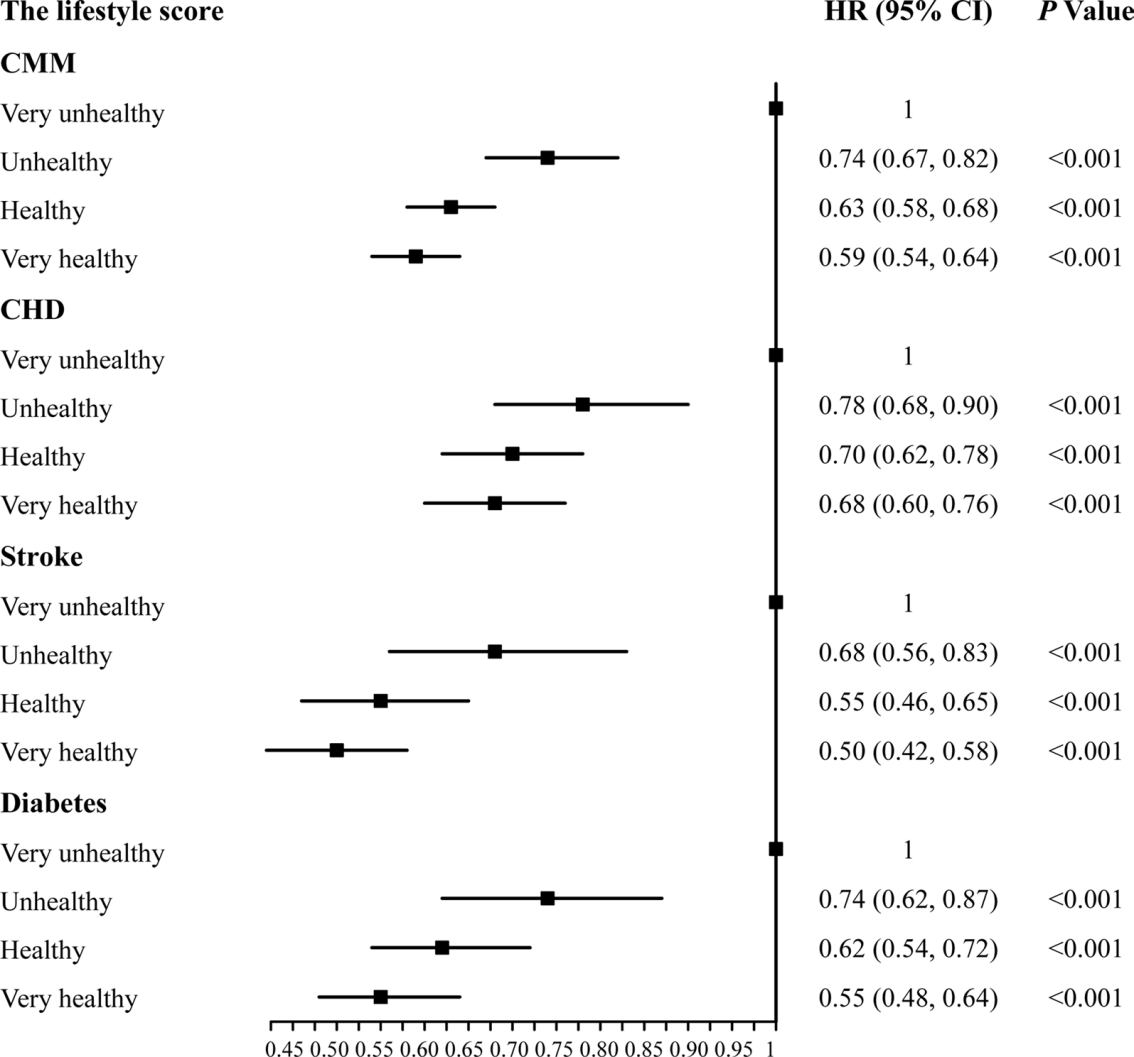
The relationship between a healthy lifestyle and cardiometabolic multimorbidity. We used four healthy lifestyle factors to estimate the lifestyle score, 0 points: current smoking, excessive drinking, less than two types of a healthy diet, no regular physical activity; 1 point, including non-current smoking, non-excessive drinking, more than 2 types of a healthy diet, regular physical activity. Then, we multiplied the binary lifestyle variables of each participant using the standardised weighted β coefficient and then summed and grouped participants into four ordered categories: very unhealthy (0 ≤ scores < 0.25, reference group), unhealthy (0.25 ≤ scores < 0.50), healthy (0.50 ≤ scores < 0.75), and very healthy (0.75 ≤ scores ≤ 1). CMM was defined as the occurrence of ≥ 1 of the following: CHD (myocardial infarction or angina), stroke (ischaemic stroke, cerebral haemorrhage, or subarachnoid haemorrhage), and diabetes. We adjusted for sex, ethnicity (white or non-white), Townsend deprivation index, sedentary time, antihypertensives drugs, lipid-lowering drugs, aspirin, and the year of attending the assessment centre

When confounding factors in Model 1, 2, and 3 were adjusted and analysed, all models showed that compared with the very unhealthy group, other groups with healthier lifestyles had a lower risk of CMM, with the very healthy group having the strongest protective effect (Additional file 1: Table S2). In Model 3, compared with the very unhealthy (reference) group, the adjusted HRs of the occurrence of CMM was 0.74 (95% CI 0.67–0.82) in the unhealthy group, 0.63 (95% CI 0.58–0.68) in the healthy group, and 0.59 (95% CI 0.54–0.64) in the very healthy group (Fig. 2).

Similarly, for specific cardiometabolic diseases, the results showed that compared with the very unhealthy group, other groups with healthier lifestyles had stronger protection against specific conditions, with the very healthy group having the strongest protective effect (Additional file 1: Table S2). Compared with the very unhealthy (reference) group, the risks for CHD, stroke, and diabetes in the very healthy group was reduced by 32%, 50%, and 45%, respectively (Fig. 2).

The associations between each healthy lifestyle and CMM are presented in Table S3 (Additional file 1), the strongest having been observed for non-smoking. In Model 3, compared with the current smoking group, the risk for CMM in the non-current smoking group was reduced by 36% (HR: 0.64, 95% CI 0.60–0.68). No statistically significant effects were observed for alcohol consumption (HR: 1.02; 95% CI 0.98–1.06). Compared with the unhealthy group, regular physical activity and a healthy diet reduced the risk of CMM by 9% and 8% among patients with hypertension.

### Differences in CMM-free life expectancy

**Fig. 3:**
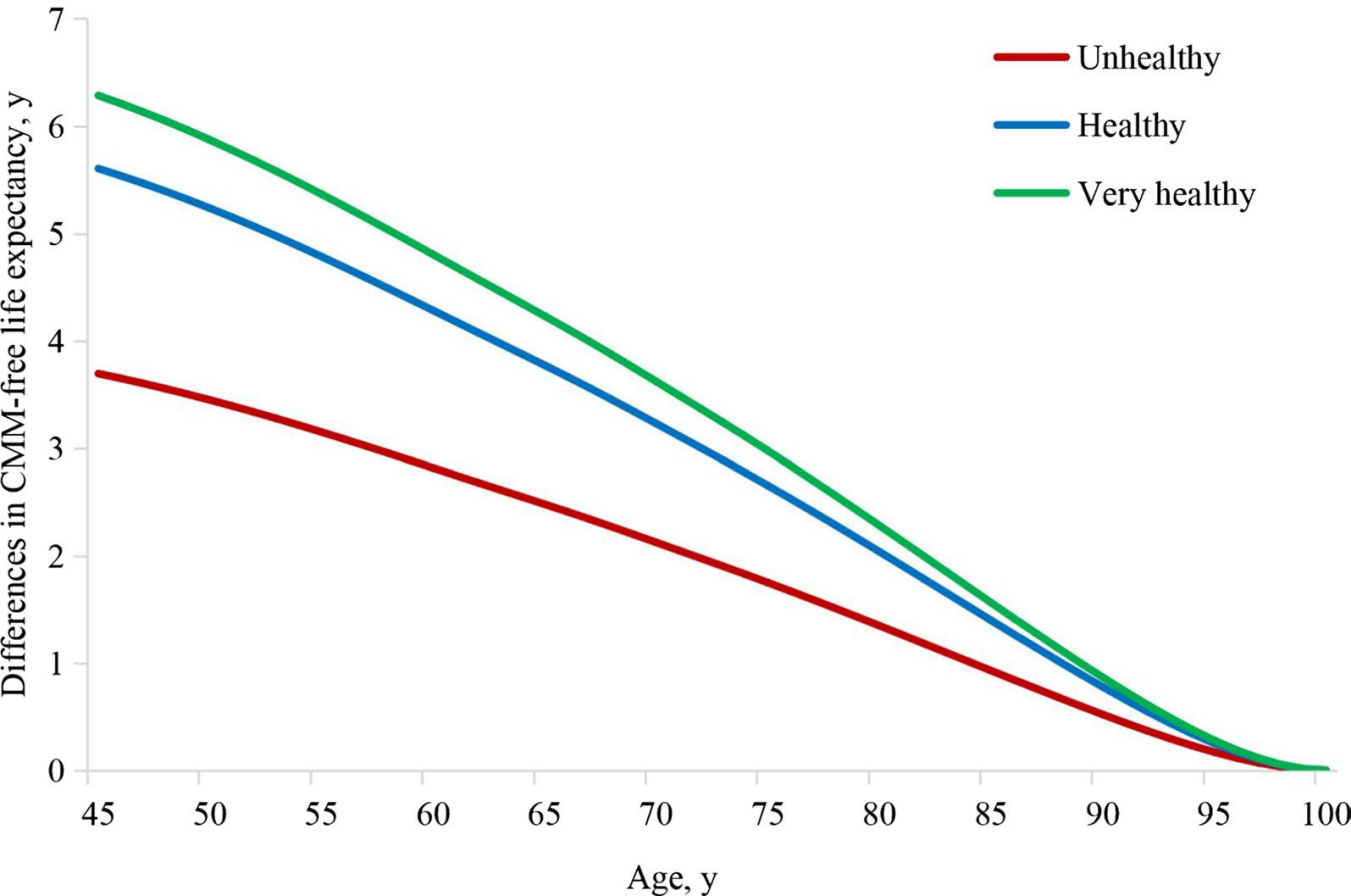
The relationship between a healthy lifestyle and the survival years difference without cardiometabolic multimorbidity. The difference in CMM-free life expectancy refers to the number of survival years difference between 45 to 100 years that hypertensive participants did not have CMM (CHD, stroke, or diabetes). We used four healthy lifestyle factors to estimate the lifestyle score. 0 points: current smoking, excessive drinking, less than two types of a healthy diet, no regular physical activity; 1 point, including non-current smoking, non-excessive drinking, more than 2 types of a healthy diet, regular physical activity. Then, we multiplied the binary lifestyle variables of each participant using the standardised weighted β coefficient and then summed and grouped participants into four ordered categories: very unhealthy (0 ≤ scores < 0.25, reference group), unhealthy (0.25 ≤ scores < 0.50), healthy (0.50 ≤ scores < 0.75), and very healthy (0.75 ≤ scores ≤ 1). We adjusted for sex, ethnicity (white or non-white), Townsend deprivation index, sedentary time, antihypertensives drugs, lipid-lowering drugs, aspirin, and the year of attending the assessment centre. *CMM* cardiometabolic multimorbidity, *CHD* coronary heart disease

Compared with the very unhealthy group, the other groups with more healthy lifestyle factors had a longer CMM-free life expectancy (Fig. 3). After adjustments for variables in Model 3, compared to the very unhealthy group, the difference in CMM-free life expectancy for the unhealthy group increased by 3.69 (95% CI 2.46–4.93) years, that for the healthy group increased by 5.60 (95% CI 4.54–6.67) years, and that for the very healthy group increased by 6.28 (95% CI 5.25–7.32) years at the age of 45 years. Corresponding estimates at the age of 65 years were 2.48 (95% CI 1.65–3.30), 3.77 (95% CI 3.05–4.49), and 4.23 (95% CI 3.53–4.93) years for the unhealthy, healthy, and very healthy groups, respectively.

### Results of subgroup analysis

Subgroup analysis was performed using Model 3 (Additional file 1: Fig. S1). No statistically significant interaction with a healthy lifestyle was shown for sex, ethnicity, sedentary time, or the use of antihypertensive drugs, lipid-lowering drugs, or aspirin (*P* ≥ 0.05). However, there were statistically significant interactions in terms of BMI and Townsend deprivation index (*P* < 0.05). In the BMI < 30 group, the risk for CMM was reduced by 46% in the very healthy group compared with the very unhealthy group. However, in the BMI ≥ 30 groups, the risk was only reduced by only 26%. Participants with a higher Townsend deprivation index (poor economic status) showed a stronger protective effect of a healthy lifestyle on the occurrence of CMM.

### Results of sensitivity analysis

Seven sensitivity analyses were conducted using Model 3, and the results did not materially change the relationship between a healthy lifestyle and the risk for CMM in patients with hypertension (Additional file 1: Fig. S2, Text S2). Using lifestyle score as a continuous variable, a one-point increase in the healthy lifestyle score reduced the risk for CMM by 9% (HR: 0.91, 95% CI 0.89–0.93).

## Discussion

Our results showed that combined healthy lifestyle factors were associated with a lower risk of CMM in hypertensive patients. This result remained statistically significant after adjusting for sex, ethnicity, Townsend deprivation index, BMI, sedentary time, antihypertensives drugs, lipid-lowering drugs, aspirin, and year of attending the assessment centre. Compared with the very unhealthy group, the very healthy group had a 41% reduction in the risk of occurring CMM in hypertensive patients and a 32–50% reduction in the risk for specific cardiometabolic diseases. The combined healthy lifestyle extended the differences in CMM-free life expectancy. These findings have significant implications for hypertensive individuals and clinical and public health departments, as the results proved that adhering to a healthier lifestyle still has apparent benefits in patients with hypertension.

A healthy lifestyle can reduce mortality and extend life expectancy, which is the cornerstone of reducing the global burden of disease [23–25]. However, the association between a healthy lifestyle and multimorbidity has remained inconsistent [26–33] Most studies suggest that healthy lifestyle factors have a protective effect against the occurrence of CMM, while a few studies have observed no association possibly owing to small sample sizes [30], short follow-up time [30], older population [30], and large variation among number and the types of multimorbidity measured [1, 11, 28, 32]. Moreover, these studies were based on the general population and focused on the primary prevention of multimorbidity. In contrast, our study was based on participants with hypertension, emphasizing the secondary prevention effect of a healthy lifestyle on hypertension. Furthermore, the definition of multimorbidity in our study is more specific, with a focus on CMMs including hypertension, CHD, diabetes, and stroke. Since these four chronic diseases have similar overall pathophysiologic risk profiles, patients with CMM are more likely to have similar self-management plans [34].

Our study on the relationship between a combined healthy lifestyle and CMM is consistent with the previous studies [11, 13, 35, 36], as the risk of occurring CMM was reduced by 41% in the very healthy group compared with the very unhealthy group. Similarly, a prospective Chinese study investigated the associations between five healthy lifestyles, cardiometabolic disease, and multimorbidity showing that for each additional high-risk lifestyle factor, the risk of progression from healthy to cardiometabolic disease and from cardiometabolic disease to CMM increased by 20% and 14%, respectively [11]. Another study showed that compared with the no-high-risk lifestyle group, the four high-risk lifestyle groups (current smoking, physical inactivity, poor diet, and heavy drinking) had a 64% and 209% increase in the risks of cardiometabolic diseases and CMM, respectively [13]. Therefore, multiple studies suggest the adoption of combined lifestyle factors to reduce the risk of cardiometabolic multimorbidity.

There have been many studies on the associations between lifestyle factors and specific cardiometabolic diseases. In our investigation of the relationship between a combination of healthy lifestyle factors and specific cardiometabolic diseases such as CHD, stroke, and diabetes, results showed that combined healthy lifestyle factors had a protective effect against the occurrence of specific cardiometabolic diseases, which is consistent with other studies [37–41]. A combined healthy lifestyle including non-smoking, consuming small amounts of alcohol, regular physical activity, healthy diet, and normal weight, can reduce the risk of cardiovascular disease, stroke, and diabetes by approximately 66%, 60%, and 75% [39, 41], respectively, whereas our study showed that combined healthy lifestyles in participants with hypertension can reduce the risk of CHD, stroke, and diabetes by 32%, 50%, and 45%, respectively. These results showed a protective effect of lifestyle interventions against the occurrence of specific cardiometabolic diseases that was lower than that reported in previous studies. This may have been due to differences in calculations for the combined lifestyles. Unlike previous studies, which used a simple cumulative scoring method, our study used a weighted healthy lifestyle assessment. Furthermore, assigning different weights to different lifestyles reflects an inconsistent risk of cardiometabolic diseases. Additionally, in contrast to the general population, participants with hypertension are more prone to heart, brain, kidney, and other target organ damage [8, 42]. These patients inherently have various pathophysiological mechanisms such as sodium intake, insulin resistance, obesity, genetics, endothelial dysfunction, oxidative stress, renin–angiotensin–aldosterone system dysfunction, and sympathetic nervous system dysfunction, that contribute to elevated blood pressure [43–46]. Although participants with hypertension receive more education and pay more attention to healthy lifestyles than the general population, they are unable to change the essential characteristics of their existing hypertensive diseases. However, our results showed that even for those with pre-existing diseases, adherence to a healthy lifestyle still protects against the occurrence of other cardiometabolic diseases. Our study has important clinical significance and social value for the prevention and management of CHD, stroke, and diabetes.

For this study, we chose the four traditional modifiable lifestyles (smoking, alcohol consumption, physical activity, and diet) that are generally presumed to be related to multimorbidity [47, 48]. Our findings that non-smoking, physical activity, and a healthy diet had the ability to reduce the risk for CMM individually and that non-smoking had the greatest protective effect against the occurrence for CMM were consistent with those of some existing studies on the general population [13, 16, 32]. However, there were no significant differences in alcohol consumption. Generally, reduction of alcohol consumption for the prevention of hypertension is controversial [49–51]. Alcohol consumption in patients with hypertension is reportedly inversely associated with cardiovascular events and all-cause mortality [52]; our findings were inconsistent with this, possibly due to the assessment of alcohol consumption, the outcomes of interest, and the large proportion of Caucasian patients in our study. Overall, our results highlighted the importance of smoking cessation, a comparatively feasible and economical measure for promoting population health and reducing the disease burden.

Hypertension is the most significant cause of early death globally [53] and the first leading risk factor for global disease burden [54, 55]. When patients with hypertension occur CMM, it further increases mortality and shortens life expectancy [1–3, 5]. Since our results showed that a combination of healthy lifestyle may reduce the risk for CMM in patients with hypertension, any practices and policies that support the adoption of healthy lifestyle factors are encouraged as they will improve the overall health of the population.

### Strengths and limitations

This study has several strengths. First, this is a large-sample prospective cohort study, and second, we used the flexible parameter Royston-Parmar proportional hazard model to estimate the β coefficient of each healthy lifestyle factor. Compared with the previously combined lifestyle studies [11, 25, 37, 41], we accessed the weighted healthy lifestyle scores of four traditional risk factors. Thus, this method reflected the differences between different healthy lifestyles in the occurrence of CMM.

This study also has some limitations. First, the adoption of healthy lifestyle factors is based on self-reported baseline data, which may change over time. However, without intervention, most people are not likely to change their healthy lifestyles after a newly diagnosed disease [56]. In addition, there might also have been a few participants who made lifestyle changes such as quitting smoking, increasing exercise, and eating healthier due to elevated blood lipids and blood pressure. Because this portion of the population was part of the unhealthy group in our study, our results may have also slightly underestimated the protective effect of healthy lifestyle factors. Second, lifestyle data is self-reported, and recalling bias and social desirability bias might have appeared, although most large epidemiological studies are based on self-reported questionnaires. Due to social desirability bias, people tend to overreport socially desirable behaviours and underreport undesirable behaviours [57, 58]. This may have led to some participants with unhealthy habits being mistakenly placed in healthy groups. The protective effect of healthy lifestyle factors might also have been underestimated. Third, we did not include other healthy lifestyles such as sleep duration and quality, sedentary time, or BMI, but instead adjusted the sedentary time and BMI in our analysis. Fourth, this was an observational study and did not prove a causal link. Fifth, the study was based on UK Biobank database, of which 97.3% of the reporting population is Caucasian. This may have affected the generalisability but not the internal validity of our results. Sixth, whether the blood pressure of hypertensive patients is controlled to meet the standard is an important factor affecting the occurrence of CMM. However, since the first blood pressure measurement was included in this study, blood pressure follow-up data were lacking. We did not perform a stratified analysis of whether blood pressure was well controlled to explore the relationship between a healthy lifestyle and CMM; thus, future studies are required to further establish a prospective cohort study for hypertension and incorporate blood pressure control into the research analysis.

## Conclusions

In conclusion, our study suggests that combined healthy lifestyle factors may reduce the risk for CMM in patients with hypertension and lower the overall burden of the disease. Thus, policies and practices that support healthy lifestyles and improve overall health are encouraged.

## Supplementary Information

**Additional file 1: Figure S1**. The relationship between a healthy lifestyle and CMM in subgroup analyses. **Figure S2.** The relationship between a healthy lifestyle and CMM in seven sensitivity analyses. **Figure S3.** The frequency distribution graph of the weighted healthy lifestyle score for the whole population. **Figure S4.** The frequency distribution graph of the weighted healthy lifestyle score for 2/3 of the population. **Figure S5.** The frequency distribution graph of the sum healthy lifestyle score for the whole population. **Table S1.** Cardiometabolic diseases definitions. **Table S2.** The association between a healthy lifestyle and cardiometabolic multimorbidity. **Table S3.** The association between each healthy lifestyle and cardiometabolic multimorbidity. **Text S1.** Alcohol consumption calculation method. **Text S2.** How the weighted healthy lifestyle scores and summed lifestyle scores were calculated.

## Data Availability

The data that support the findings of this study are available from UK Biobank but restrictions apply to the availability of these data, which were used under license for the current study, and so are not publicly available. Data are however available from the authors upon reasonable request and with permission of UK Biobank.

## Abbreviations

BMI: Body mass index
CHD: Coronary heart disease
CI: Confidence interval
CMM: Cardiometabolic multimorbidity
HR: Hazard ratio
RMST: Restricted mean survival time
