# Supplementary material for: Association between healthy lifestyle and the occurrence of cardiometabolic multimorbidity in hypertensive patients: a prospective cohort study of UK Biobank
Source: Cardiovasc Diabetol. 2022 Oct 1;21:199. doi: 10.1186/s12933-022-01632-3 (PMC9526960; doi:10.1186/s12933-022-01632-3)
Supplement: Supplementary file 1 — Additional file 1: Figure S1. The relationship between a healthy lifestyle and CMM in subgroup analyses. Figure S2. The relationship between a healthy lifestyle and CMM in seven sensitivity analyses. Figure S3. The frequency distribution graph of the weighted healthy lifestyle score for the whole population. Figure S4. The frequency distribution graph of the weighted healthy lifestyle score for 2/3 of the population. Figure S5. The frequency distribution graph of the sum healthy lifestyle score for the whole population. Table S1. Cardiometabolic diseases definitions. Table S2. The association between a healthy lifestyle and cardiometabolic multimorbidity. Table S3. The association between each healthy lifestyle and cardiometabolic multimorbidity. Text S1. Alcohol consumption calculation method. Text S2. How the weighted healthy lifestyle scores and summed lifestyle scores were calculated. [file 12933_2022_1632_MOESM1_ESM.pdf]

Additional File 1

List of contents:

Figure S1. The relationship between a healthy lifestyle and CMM in subgroup analyses.

Figure S2. The relationship between a healthy lifestyle and CMM in seven sensitivity analyses.

Figure S3. The frequency distribution graph of the weighted healthy lifestyle score for the whole population.

Figure S4. The frequency distribution graph of the weighted healthy lifestyle score for 2/3 of the population.

Figure S5. The frequency distribution graph of the sum healthy lifestyle score for the whole population.

Table S1. Cardiometabolic diseases definitions.

Table S2. The association between a healthy lifestyle and cardiometabolic multimorbidity.

Table S3. The association between each healthy lifestyle and cardiometabolic multimorbidity.

Text S1. Alcohol consumption calculation method

Text S2. How the weighted healthy lifestyle scores and summed lifestyle scores were calculated

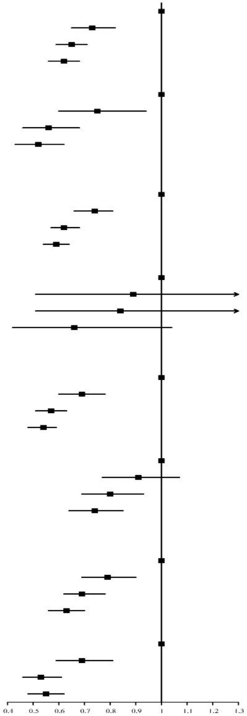

The lifestyle score	HR (95% CI)	*P* Value
**Sex**		**0.192**
**Male**		
Very unhealthy	1	
Unhealthy	0.73 (0.65, 0.82)	
Healthy	0.65 (0.59, 0.71)	
Very healthy	0.62 (0.56, 0.68)	
**Female**		
Very unhealthy	1	
Unhealthy	0.75 (0.60, 0.94)	
Healthy	0.56 (0.46, 0.68)	
Very healthy	0.52 (0.43, 0.62)	
**Ethnicity**		**0.739**
**White**		
Very unhealthy	1	
Unhealthy	0.74 (0.66, 0.81)	
Healthy	0.62 (0.57, 0.68)	
Very healthy	0.59 (0.54, 0.64)	
**Non−white**		
Very unhealthy	1	
Unhealthy	0.89 (0.51, 1.54)	
Healthy	0.84 (0.51, 1.36)	
Very healthy	0.66 (0.42, 1.04)	
**BMI**		**0.010**
**BMI<30**		
Very unhealthy	1	
Unhealthy	0.69 (0.60, 0.78)	
Healthy	0.57 (0.51, 0.63)	
Very healthy	0.54 (0.48, 0.59)	
**BMI≥30**		
Very unhealthy	1	
Unhealthy	0.91 (0.77, 1.07)	
Healthy	0.80 (0.69, 0.93)	
Very healthy	0.74 (0.64, 0.85)	
**Townsend deprivation index**		**0.011**
**≤0**		
Very unhealthy	1	
Unhealthy	0.79 (0.69, 0.90)	
Healthy	0.69 (0.62, 0.78)	
Very healthy	0.63 (0.56, 0.70)	
**>0**		
Very unhealthy	1	
Unhealthy	0.69 (0.59, 0.81)	
Healthy	0.53 (0.46, 0.61)	
Very healthy	0.55 (0.48, 0.62)	

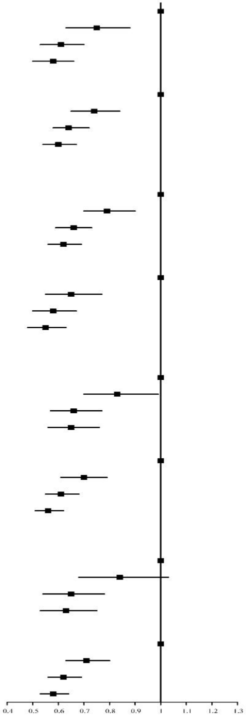

The lifestyle score	HR (95% CI)	*P* Value
**Sedentary time**		**0.918**
**Short(<5h)**		
Very unhealthy	1	
Unhealthy	0.75 (0.63, 0.88)	
Healthy	0.61 (0.53, 0.70)	
Very healthy	0.58 (0.50, 0.66)	
**Long (≥5h)**		
Very unhealthy	1	
Unhealthy	0.74 (0.65, 0.84)	
Healthy	0.64 (0.58, 0.72)	
Very healthy	0.60 (0.54, 0.67)	
**Antihypertensive drugs**		**0.291**
**YES**		
Very unhealthy	1	
Unhealthy	0.79 (0.70, 0.90)	
Healthy	0.66 (0.59, 0.73)	
Very healthy	0.62 (0.56, 0.69)	
**NO**		
Very unhealthy	1	
Unhealthy	0.65 (0.55, 0.77)	
Healthy	0.58 (0.50, 0.67)	
Very healthy	0.55 (0.48, 0.63)	
**Lipid−lowering drugs**		**0.100**
**YES**		
Very unhealthy	1	
Unhealthy	0.83 (0.70, 0.99)	
Healthy	0.66 (0.57, 0.77)	
Very healthy	0.65 (0.56, 0.76)	
**NO**		
Very unhealthy	1	
Unhealthy	0.70 (0.61, 0.79)	
Healthy	0.61 (0.55, 0.68)	
Very healthy	0.56 (0.51, 0.62)	
**Aspirin**		**0.329**
**YES**		
Very unhealthy	1	
Unhealthy	0.84 (0.68, 1.03)	
Healthy	0.65 (0.54, 0.78)	
Very healthy	0.63 (0.53, 0.75)	
**NO**		
Very unhealthy	1	
Unhealthy	0.71 (0.63, 0.80)	
Healthy	0.62 (0.56, 0.69)	
Very healthy	0.58 (0.53, 0.64)	

Figure S1. The relationship between a healthy lifestyle and CMM in subgroup analyses

We used four healthy lifestyle factors to estimate the lifestyle score, 0 points: current smoking, excessive drinking, less than two types of a healthy diet, no regular physical activity; 1 point, including non-current smoking, non-excessive drinking, more than two types of a healthy diet, regular physical activity. Then, we multiplied the binary lifestyle variables of each participant using the standardised weighted β coefficient and then summed and grouped participants into four ordered categories: very unhealthy ($0 \leq$ scores < 0.25, reference group), unhealthy ($0.25 \leq$ scores < 0.50), healthy ($0.50 \leq$ scores < 0.75), and very healthy ($0.75 \leq$ scores ≤ 1). CMM was defined as the occurrence of ≥ 1 of the following: CHD (myocardial infarction or angina), stroke (ischaemic stroke, cerebral haemorrhage, or subarachnoid haemorrhage), and diabetes. We adjusted for sex, ethnicity (white or non-white), Townsend deprivation index, sedentary time, antihypertensives drugs, lipid-lowering drugs, aspirin, and the year of attending the assessment centre.

Abbreviations: HR, hazard ratio; CI, confidence interval; CMM, cardiometabolic multimorbidity; CHD, coronary heart disease

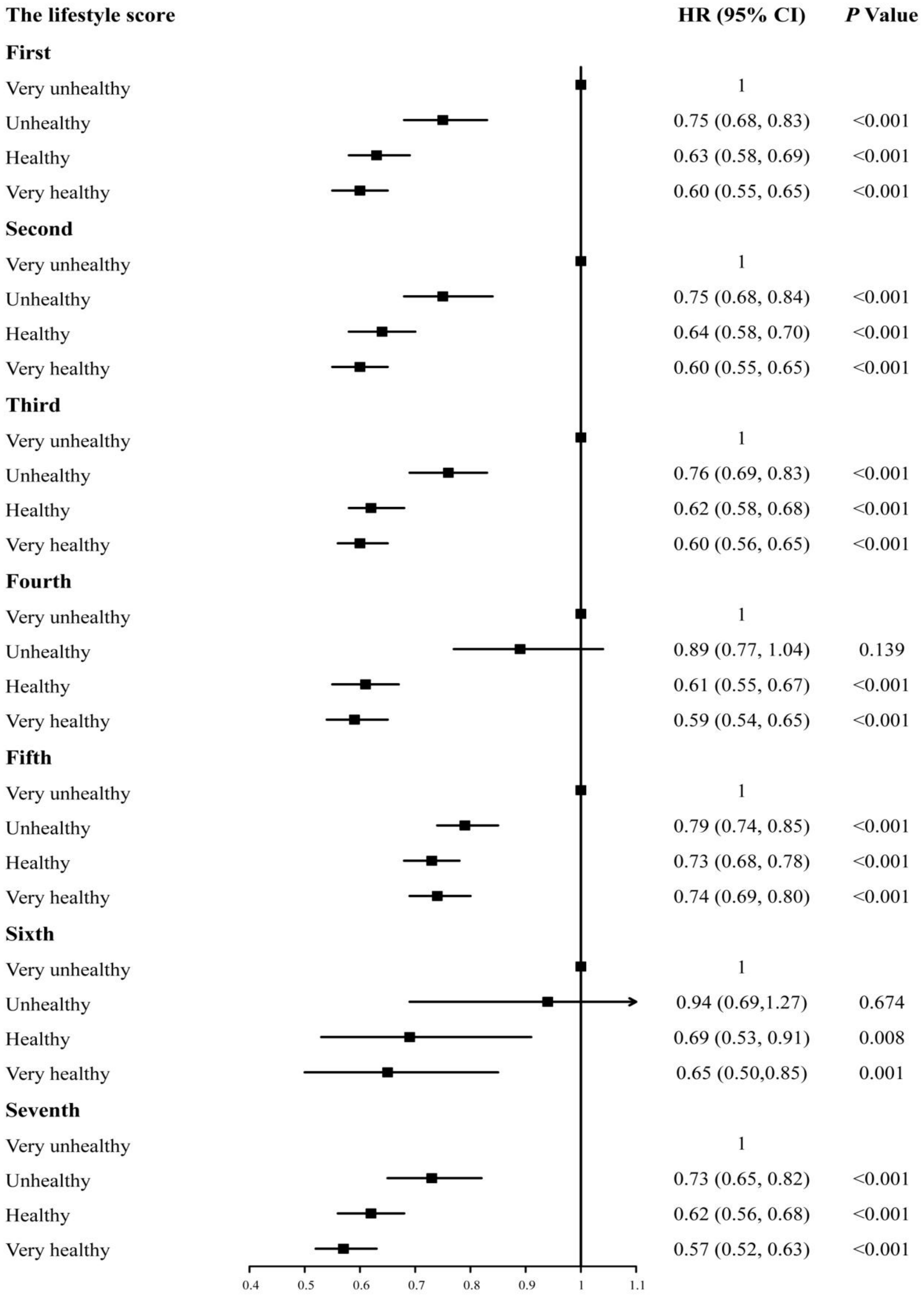

Figure S2. The relationship between a healthy lifestyle and CMM in seven sensitivity analyses

Abbreviations: CI, confidence interval; CMM, cardiometabolic multimorbidity

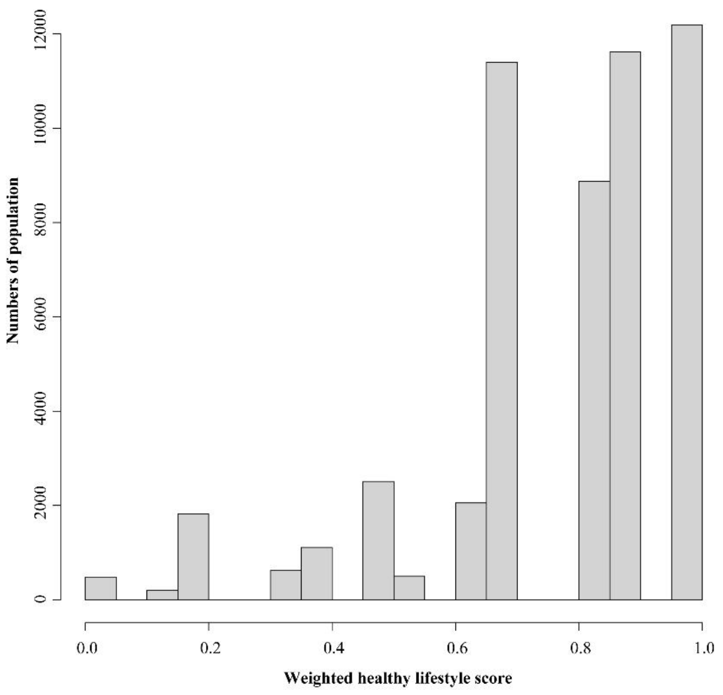

Figure S3. The frequency distribution graph of the weighted healthy lifestyle score for the whole population.

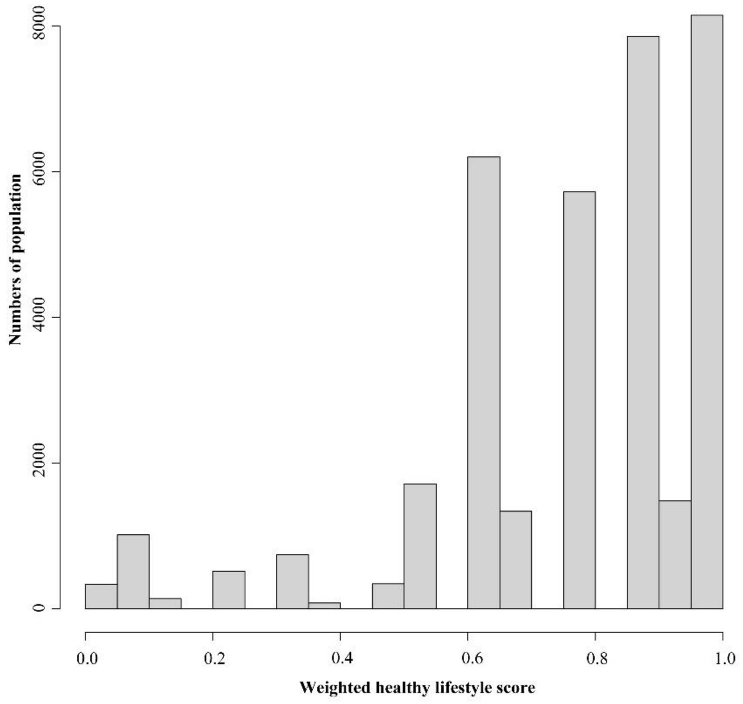

Figure S4. The frequency distribution graph of the weighted healthy lifestyle score for 2/3 of the population

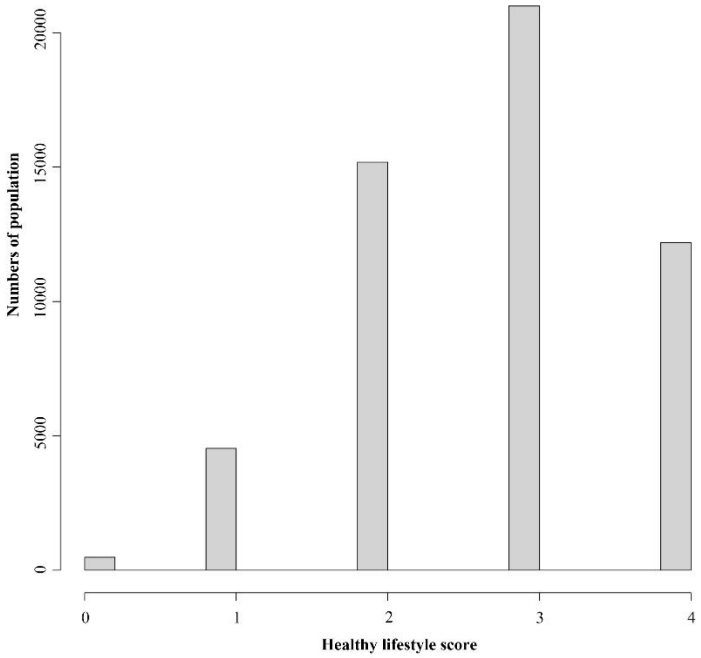

Figure S5. The frequency distribution graph of the sum healthy lifestyle score for the whole population.

Table S1. Cardiometabolic diseases definitions

Cardiometabolic diseases	Self-reported Information	ICD-9 information	ICD-10 information	OPCS-4 information
Diabetes	2443 (1), 2976, 6153 (3), 6177 (3), 20002 (1220, 1222, 1223), 20008, 20009	41271 (250, 3572, 3620) 41281	41270 (E10-E14, G590, G632, H280, H360, M142, N083) 41280, 130714, 130712, 130710, 130708, 130706, 130715, 130713, 130711, 130709, 130707	
Hypertension	2966, 6150 (4), 6153 (2), 6177 (2), 20002 (1065, 1072), 20008, 20009	41271 (401-405) 41281	41270 (I10-I13, I15, O10), 41280, 131292, 131290, 131288, 131286, 131294, 132180, 131295, 131293, 131291, 131289, 131287, 132181	
CHD	6150 (1, 2), 3894, 3627, 20004 (1070, 1095, 1523), 20002 (1074, 1075), 20008, 20009, 20010, 20011	41271 (410-414), 41281	41270 (I20-I25, Z951, Z955), 41280, 131296, 131298, 131300, 131302, 131304, 131306, 131307, 131305, 131303, 131301, 131299, 131297	41272 (K40-K46, K49, K50, K75), 41282
Stroke	6150 (3), 4056, 20002 (1081, 1491, 1583, 1086), 20008, 20009, 20010, 20011	41271 (3361, 3623, 430, 431, 4329, 4330, 4331, 4332,4333, 4338, 4339, 434, 436), 41281	41270 (I60, I61, I629, I63, I64, I678, I690, I693, G951, H341, H342, S066), 41280, 131378, 131376, 131374, 131372, 131370, 131368, 131366, 131364, 131362, 131360, 131180, 131379, 131377, 131375, 131373, 131371, 131369, 131367, 131365, 131363, 131361, 131181	41272 (A052-A054, L351, L353, L343), 41282

Abbreviations: CHD, coronary heart disease; ICD-9: International Classification of Disease version 9; ICD-10: International Classification of Disease version 10; OPCS-4: Office of Population Censuses and Surveys Classification of Interventions and Procedures, version 4.

Table S2. The association between a healthy lifestyle and cardiometabolic multimorbidity

The lifestyle score	Model 1		Model 2		Model 3	
	HR (95% CI)	*P* Value	HR (95% CI)	*P* value	HR (95% CI)	*P* Value
**CMM**						
Very unhealthy	1		1		1	
Unhealthy	0.77 (0.69, 0.85)	< 0.001	0.74 (0.67, 0.82)	<0.001	0.74 (0.67, 0.82)	<0.001
Healthy	0.63 (0.58, 0.69)	<0.001	0.63 (0.58, 0.68)	<0.001	0.63 (0.58, 0.68)	<0.001
Very healthy	0.56 (0.52, 0.61)	<0.001	0.59 (0.54, 0.64)	<0.001	0.59 (0.54, 0.64)	<0.001
**CHD**						
Very unhealthy	1		1		1	
Unhealthy	0.79 (0.69, 0.90)	<0.001	0.79 (0.68, 0.90)	<0.001	0.78 (0.68, 0.90)	<0.001
Healthy	0.69 (0.61, 0.77)	<0.001	0.70 (0.62, 0.78)	<0.001	0.70 (0.62, 0.78)	<0.001
Very healthy	0.65 (0.58, 0.72)	<0.001	0.68 (0.61, 0.76)	<0.001	0.68 (0.60, 0.76)	<0.001
**Stroke**						
Very unhealthy	1		1		1	
Unhealthy	0.65 (0.54, 0.79)	<0.001	0.68 (0.56, 0.83)	<0.001	0.68 (0.56, 0.83)	<0.001
Healthy	0.52 (0.44, 0.61)	<0.001	0.55 (0.46, 0.64)	<0.001	0.55 (0.46, 0.65)	<0.001
Very healthy	0.47 (0.40, 0.55)	<0.001	0.50 (0.42, 0.58)	<0.001	0.50 (0.42, 0.58)	<0.001
**Diabetes**						
Very unhealthy	1		1		1	
Unhealthy	0.85 (0.72, 1.00)	0.062	0.74 (0.62, 0.87)	<0.001	0.74 (0.62, 0.87)	<0.001
Healthy	0.68 (0.59, 0.79)	<0.001	0.62 (0.54, 0.72)	<0.001	0.62 (0.54, 0.72)	<0.001
Very healthy	0.52 (0.45, 0.60)	<0.001	0.55 (0.48, 0.64)	<0.001	0.55 (0.48, 0.64)	<0.001

Model 1: adjusted for sex, ethnicity (white, non-white). Model 2: additionally adjusted for Townsend deprivation index, body mass index, sedentary time. Model 3: additionally adjusted for antihypertensive drugs use, lipid-lowering drugs use, aspirin use, and the year of attending assessment centre. Abbreviations: CI, confidence interval; CMM: cardiometabolic multimorbidity; CHD: coronary heart disease; HR: hazard ratio.

Table S3. The association between individual healthy lifestyle and cardiometabolic multimorbidity

Lifestyle category	Model 1		Model 2		Model 3	
	HR (95% CI)	*P* Value	HR (95% CI)	*P* value	HR (95% CI)	*P* Value
Non-current smoking	0.63 (0.60, 0.68)	<0.001	0.64 (0.60, 0.68)	<0.001	0.64 (0.60, 0.68)	<0.001
Non-excessive drinking	0.99 (0.95, 1.03)	0.500	1.00 (0.97, 1.05)	0.669	1.02 (0.98, 1.06)	0.420
Physical activity	0.85 (0.81, 0.89)	<0.001	0.92 (0.87, 0.96)	0.001	0.91 (0.87, 0.96)	<0.001
Healthy diet	0.88 (0.85, 0.92)	<0.001	0.92 (0.88, 0.96)	<0.001	0.92 (0.88, 0.96)	<0.001

Model 1: adjusted for sex, ethnicity (white, non-white). Model 2: additionally adjusted for Townsend deprivation index, body mass index, sedentary time. Model 3: additionally adjusted for antihypertensive drugs use, lipid-lowering drugs use, aspirin use, and the year of attending assessment centre. Abbreviations: CI, confidence interval; HR: hazard ratio.

Text S1: Alcohol consumption calculation method

For alcohol consumption, the participant was asked in touchscreen questionnaires. The type of alcohol consumption average weekly consumed is based on the following five aspects:

1) glasses of red wine (There are six glasses in an average bottle).

2) glasses of white wine or champagne.

3) pints of beer or cider (Including bitter, lager, stout, ale, and Guinness).

4) measures of spirits or liqueurs (There are 25 standard measures in a normal-sized bottle; spirits include drinks such as whisky, gin, rum, vodka, and brandy).

5) glasses of fortified wine (Fortified wines include drinks such as sherry, port, and vermouth).

Since alcohol consumption differs from alcohol content, each alcohol consumption was converted into equivalent standard units. One unit equals 10 ml of pure alcohol. Converting volumes to units was based on the NHS guidelines and the Office for National Statistics survey data. The total weekly alcohol consumption is the sum of the above five types of alcohol.

The total weekly alcohol consumption of equivalent standard units = 1.5 × glasses of red wine + 1.5×glasses of white wine or champagne + 2 × pints of beer or cider + 1 × measures of spirits or liqueurs + 1× glasses of fortified wine

Text S2. How the weighted healthy lifestyle scores and summed lifestyle scores were calculated

A. The β value of main research the main research

Healthy lifestyle factor in the model	β coefficient	Weighted β coefficient
Non-current smoking (Yes vs No)	-0.446801	0.498635
Non-excessive drinking (Yes vs No)	-0.1302881	0.145403
Regular physical activity (Yes vs No)	-0.1416795	0.1581159
Healthy diet (Yes vs No)	-0.1772797	0.1978461
Total	-0.8960482	1

Non-excessive drinking: We measured weekly consumption of red or white wine, beer, cider, spirits, liqueurs, and fortified wine, and converted them into equivalent standard units according to UK National Health Service guidelines and Office for National Statistics survey data (Additional File 1: Text S1). Participants were grouped into those with excessive drinking (≥ 14 units per week) and those with non-excessive drinking (< 14 units per week)

Regular physical activity: 150 minutes of walking or moderate activity per week or 75 minutes of vigorous activity

Healthy diet: one point for more than two of the following three items, otherwise 0 points: 1) Total fruit and vegetable intake: ≥ 4.5 pieces or servings per day (1 serving being 3 tablespoons); 2) total fish intake ≥ 2 times per week; 3) red meat intake ≤ 5 times per week and processed meat intake ≤ 2 times per week.

The weighted β coefficient was estimated by the flexible parameter Royston-Parmar proportional hazard model. It included all four lifestyle factors and the occurrence of cardiometabolic multimorbidity as the outcome. The standardised weighted β coefficient = the β coefficient of each healthy lifestyle factor/sum of the β coefficients We multiplied the binary lifestyle variables of each participant by the standardized weighted β coefficient. The frequency distribution graph is shown in Fiugre S3. The healthy lifestyle score ranges from very unhealthy (0) to very healthy (1)

B. The β value of a random one-third of the population

Healthy lifestyle factor in the model	β coefficient	Weighted β coefficient
Non-current smoking (Yes vs No)	-0.4061206	0.5131746
Non-excessive drinking (Yes vs No)	-0.1131513	0.1429782
Regular physical activity (Yes vs No)	-0.07533149	0.09518897
Healthy diet (Yes vs No)	-0.1967854	0.2486583
Total	-0.7913888	1

The frequency distribution graph of the weighted healthy lifestyle score for 2/3 of the population is shown in Fiugre S4.

C. The sum healthy lifestyle score

We summed up the four lifestyle scores, ranging from 0 to 4. Participants who scored 0 and 1 were categorised into the very unhealthy group, while those who scored 2, 3, and 4 were categorised into the unhealthy, healthy, and very healthy groups, respectively. The frequency distribution graph of the sum healthy lifestyle score for the whole population is shown in Figure S5.
